# Decreased diabetes-induced glycemic impairment in WKY and SHR involves enhanced skeletal muscle *Slc2a4*/GLUT4 expression

**DOI:** 10.1186/1758-5996-6-97

**Published:** 2014-09-10

**Authors:** Ana Barbara Alves-Wagner, Robinson Sabino-Silva, Raquel S Campello, Rosana C Mori, Ubiratan F Machado

**Affiliations:** Department of Physiology and Biophysics, Institute of Biomedical Sciences, University of Sao Paulo, Sao Paulo, SP Brazil; Institute of Biomedical Sciences (ICBIM), Federal University of Uberlandia (UFU), Uberlandia, MG Brazil

**Keywords:** Hypertension, Soleus, EDL, Glycemic homeostasis, Hyperglycemia

## Abstract

**Background:**

Hypertension has been associated to diabetes, and participates in the development of diabetic complications. The spontaneously hypertensive rat (SHR) is the gold standard model for the study of hypertension, and experimental diabetes has been currently investigated in SHR. Wistar-Kyoto rat is usually taken as control for SHR, however, regarding the glycemic homeostasis, WKY may be similar to SHR, when compared to the standard Wistar rat, importantly affecting the interpretation of data. *Slc2a4* gene, which encodes the GLUT4 protein, is expressed in insulin-sensitive tissues, such as muscle cells and adipocytes, and alteration in *Slc2a4*/GLUT4 expression is inversely related to glycemic levels. We investigated the effect of diabetes on the expression of *Slc2a4*/GLUT4 and glycemic control in Wistar-Kyoto and SHR.

**Findings:**

*Slc2a4* mRNA (Northern-blotting) and GLUT4 protein (Western-blotting) were investigated in skeletal muscles (soleus and extensor digitorum longus) of Wistar, Wistar-Kyoto and SHR, rendered or not diabetic for 1 month. Non-diabetic SHR shows hyperinsulinemia, and unaltered GLUT4 expression. The hyperglycemia was significantly attenuated in diabetic Wistar-Kyoto and SHR, compared to that observed in diabetic Wistar, although all of them presented the same hypoinsulinemic levels. Besides, diabetes significantly reduced *Slc2a4*/GLUT4 in Wistar, as expected; however, that was not observed in diabetic Wistar-Kyoto and SHR.

**Conclusions:**

Non-diabetic SHR is insulin resistant, despite unaltered GLUT4 expression. Diabetic Wistar-Kyoto and diabetic SHR presented high *Slc2a4*/GLUT4 expression in skeletal muscle, as compared to diabetic Wistar. This *Slc2a4*/GLUT4 regulation does not depend on insulin level and possibly protects the WKY and SHR from severe glycemic impairment.

## Background

The spontaneously hypertensive rat (SHR) strain was developed by Okamoto and Aoki [1], and it is the most studied animal model for human essential hypertension. As a result of selection for increased blood pressure prone Wistar-Kyoto rats [1], researchers usually take the normotensive Wistar-Kyoto rats (WKY) as the controls for SHR. In this context, although the Wistar-Kyoto rats are normotensive, some of their features may alter due to biological variability [2], besides being different from the standard Wistar rat [3, 4].

The glucose transporter GLUT4, encoded by the solute carrier 2A4 (*Slc2a4*) gene, is the insulin-regulatable glucose transporter, and confers to the skeletal muscle and adipose tissue a fundamental role in the glycemic homeostasis [5]. As compared to Wistar rats, WKY and SHR are glucose intolerant, and show some age-dependent changes in *Slc2a4* mRNA and GLUT4 protein in gastrocnemius and heart [3, 6].

The association of hypertension and diabetes has been highlighted since the advent of the metabolic syndrome [7]. Besides, in the development of diabetic complications such as in the nephropathy, hypertension is the major clinical factor associated [8]. Thus, the association of experimental diabetes and hypertension has been currently investigated in SHR [9–12]; meanwhile, the choice of the control animal is still controversial.

Considering the above, the present study investigated the effect of diabetes on *Slc2a4* and GLUT4 expression in skeletal muscles, as well on the glycemic homeostasis of Wistar, Wistar-Kyoto and SHR.

## Methods

### Animals

Twelve-week old male Wistar rats (W), Wistar-Kyoto rats (WKY) and spontaneously hypertensive rats (SHR) from the Animal Center of the Institute of Biomedical Sciences, University of Sao Paulo (Sao Paulo, Brazil), were rendered diabetic by alloxan injection (40 mg/kg body-weight), as previously described [10, 11]. After 4 weeks of diabetes induction, the following groups were investigated: non-diabetic Wistar (W), diabetic-Wistar (D-W), non-diabetic Wistar-Kyoto (WKY), diabetic Wistar-Kyoto (D-WKY), non-diabetic SHR (SHR) and diabetic SHR (D-SHR). The experimental protocol (#015/2008) was approved by the Ethical Committee for Animal Research of the Institute of Biomedical Sciences, University of Sao Paulo.

### Blood and urine collection and analysis

Twenty-four hour urine was collected immediately before the experiment (8:00–10:00 AM, without food restriction). The animals were anesthetized (sodium pentobarbital, 40 mg/kg body weight, i.p.) and blood samples were collected for analysis. Blood glucose concentration was measured by a glucometer (Precision QID, MediSense, Sao Paulo, SP, Brazil), urinary glucose concentration by the enzymatic-colorimetric method (Glicose Enzimatica, ANALISA Diagnostica, Belo Horizonte, BR), and plasma insulin by radioimunoassay (Coat-a-Count Insulin DPC, Los Angeles, CA, USA).

### Tissue collection and analysis

Soleus and extensor digitorum longus (EDL) muscles were harvested, and immediately processed for analysis of *Slc2a4* mRNA (Northern blotting) and GLUT4 protein (Western blotting), as previously described [13, 14]. The loading control was performed by beta actin mRNA hybridization in Northern, and by the coomassie-brilliant-blue-stained gel in Western [15].

### Data analysis

All values were reported as mean ± SEM. The comparisons were performed by one-way ANOVA, with Student Newman Keuls (SNK) as the post hoc test.

## Results

Table 1 shows the general characteristics of the rats. The body weight of non-diabetic SHR and WKY was smaller (P < 0.001 vs. W); however, the weights of both soleus and EDL muscles were similar among the groups. D-W and D-WKY had decreased body weight (P < 0.001 and P < 0.05, respectively) while in D-SHR body weight was unchanged, as compared to their respective controls. Only the EDL weight of W rats was reduced by diabetes. SHR plasma insulin was the highest; however, diabetes reduced the plasma insulin to similar values in all groups. Curiously, the degree of glycemic imbalance was much more severe in D-W than in D-SHR or D-WKY, considering the significantly (P < 0.001) higher values of glycemia, urinary volume and glucose excretion.

**Table 1:** **General characteristics of the animals**

	W	D-W	WKY	D-WKY	SHR	D-SHR
Body weight, g	341 ± 12	259 ± 7^***^	288 ± 8^***^	234 ± 15^+^	264 ± 8^***^	253 ± 12
Soleus weight, g	0.12 ± 0.007	0.11 ± 0.004	0.11 ± 0.006	0.11 ± 0.003	0.11 ± 0.005	0.11 ± 0.006
EDL weight, g	0.16 ± 0.01	0.10 ± 0.009^**^	0.14 ± 0.01	0.14 ± 0.006	0.13 ± 0.008	0.12 ± 0.01
Plasma glucose, mg/dl	136 ± 11	517 ± 29^***^	130 ± 4	239 ± 6^+++§§§^	149 ± 15	238 ± 10°°°^§§§^
Plasma insulin, μU/ml	39 ± 5.5	10 ± 2.8^**^	33 ± 3.7	11 ± 2.7^++^	56 ± 8.6^*++^	9 ± 2.3°°°
Urinary volume, ml	5.0 ± 0.8	169 ± 36^***^	1.7 ± 0.4	46 ± 7^§§§^	5.5 ± 0.7	52 ± 6^§§§^
Urinary glucose, mg/24hs	0.93 ± 0.08	340 ± 13^***^	0.18 ± 0.06	121 ± 22^+++§§§^	1.17 ± 0.5	126 ± 9°°°^§§§^

Morphometric and metabolic parameters were collected from Wistar (W), diabetic Wistar (D-W), Wistar-Kyoto (WKY), diabetic Wistar-Kyoto (D-WKY), SHR (SHR) and diabetic SHR (D-SHR). Data are mean ± SEM of 5 to 7 animals. *P < 0.05, **P < 0.01, ***P < 0.001 vs. W; ^+^P < 0.05, ^++^P < 0.01, ^+++^P < 0.001 vs. WKY; °°°P < 0.001 vs. SHR; ^§§§^P < 0.001 vs. D-W. One-way ANOVA, Student Newman Keuls (SNK) post hoc test.

**Figure 1:**
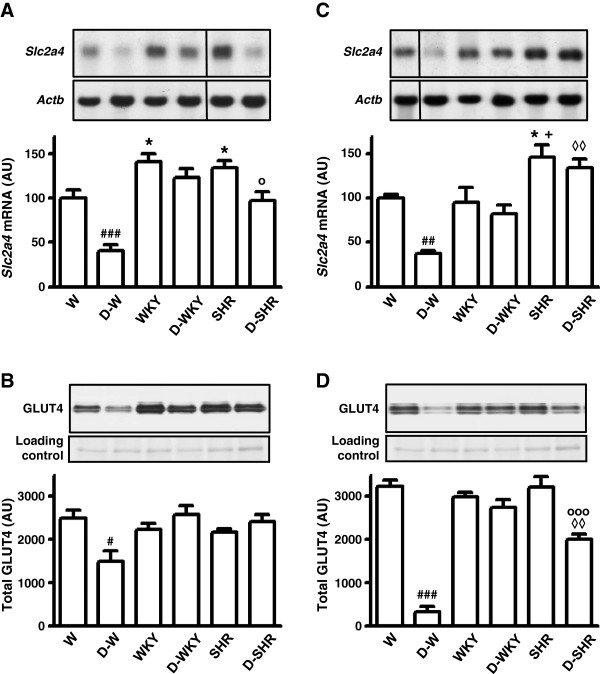
***Slc2a4*** **and GLUT4 expression in skeletal muscle.** Rats were injected with either vehicle (Wistar, W; Wistar-Kyoto, WKY and Spontaneously Hypertensive Rats, SHR) or alloxan (diabetic Wistar, D-W; diabetic Wistar-Kyoto, D-WKY and diabetic SHR, D-SHR), and studied after 4 weeks, without any treatment. Skeletal muscles were excised for analysis of soleus *Slc2a4* mRNA **(A)** and GLUT4 protein **(B)** expression and EDL *Slc2a4* mRNA **(C)** and GLUT4 protein **(D)** expression. Data are mean ± SEM of 5 to 7 animals. #P < 0.05, ##P < 0.01, ###P < 0.001 vs. other groups, *P < 0.05 vs. W, + P < 0.05 vs. WKY, ◊◊P < 0.01 vs. D-WKY, ○P < 0.05, ○○○P < 0.001 vs. SHR. One-way ANOVA, SNK post hoc test.

Concerning *Slc2a4*/GLUT4 expression, WKY and SHR showed higher *Slc2a4* mRNA content in soleus, as compared to W (P < 0.05, Figure 1A); however, the GLUT4 expression did not alter (Figure 1B). In EDL, *Slc2a4* mRNA was highest in SHR (P < 0.05 vs. W and WKY, Figure 1C), but again this difference did not reflect in GLUT4 expression (Figure 1D).

As expected, diabetes reduced (P < 0.05 to P < 0.001) *Slc2a4* mRNA and GLUT4 protein expression in soleus (Figure 1A and B) and EDL (Figure 1C and D) of D-W rats. On the other hand, diabetes did not affect *Slc2a4* or GLUT4 expression in either soleus or EDL of D-WKY rats. Similarly GLUT4 protein was unchanged in soleus of D-SHR (Figure 1B), but it decreased in EDL of D-SHR (P < 0.001 vs. SHR, Figure 1D). Importantly, the GLUT4 protein was significantly higher in D-WKY and D-SHR as compared to D-W, in both soleus and EDL.

## Discussion

Previous studies have proposed that WKY and SHR are insulin resistant, both presenting age-dependent alterations in glycemia and in the insulin response to a glucose loading [3]. Our results show SHR maintained the same basal glycemia at the expense of a significant hyperinsulinemia, a profile consistent with decreased insulin sensitivity; however, no differences in plasma glucose and insulin levels were found between WKY and W rats, suggesting they are equally sensitive to insulin. Interestingly, WKY and SHR had a ~15% reduced body weight, as compared to the W rats. This reduction must be a consequence of reduced fat mass, considering the unaltered lean mass, which can be assumed by the similar soleus and EDL weights among the groups. In both humans and mice, weight loss has been associated with increased insulin sensitivity [16]; nevertheless, the present detection that leaner SHR are insulin resistant suggests that mechanisms other than the fat mass are regulating insulin sensitivity.

Alloxan treatment renders animals diabetic with no complete destruction of beta cell [17–19]; thus, as expected, the present diabetic groups were all hypoinsulinemic. D-W rats had a severe body weight loss, as compared to D-WKY and D-SHR, what suggests some degree of protection against diabetes-induced weight loss in WKY and SHR. Surprisingly, the glycemic derangement was much worse in D-W than in D-WKY and D-SHR, and that was not related to a different degree of beta cells destruction, since basal insulinemia was similar among the diabetic groups. Thus, WKY and SHR seem to be somewhat protected against the diabetic injuries.

GLUT4 protein plays a key role in the insulin-induced glucose disposal, which is mainly performed in skeletal muscle [20]. Here in, GLUT4 expression in both soleus and EDL was unchanged among the groups. So, the insulin resistance of SHR cannot be attributed to decreased GLUT4 expression as usually observed; remaining a defect in the GLUT4 translocation as a probable cause of the reduced muscle glucose disposal. In fact impaired IRS/PI3K/Akt activation [21] in skeletal muscle and GLUT4 translocation in adipose tissue [22] have already been described in SHR. Curiously the *Slc2a4* mRNA was increased in soleus from WKY and SHR and in EDL from SHR as well, despite the unchanged GLUT4 protein, pointing out a posttranscriptional regulation of this gene. During fasting, enhancing effect of beta-adrenergic activity on *Slc2a4* mRNA expression in soleus and EDL rat muscles has been suggested [14]. Since increased beta-adrenergic activity is characteristic of SHR [23], this could explain the increased *Slc2a4* expression observed in these rats. Nevertheless, it is difficult to explain the similar regulation observed in WKY, since there is no clear report comparing peripheral sympathetic activity of WKY and Wistar rats.

Diabetes decreases *Slc2a4* and GLUT4 expression in soleus, EDL and gastrocnemius muscles, as well as in white adipose tissue [19, 24], and that was presently confirmed in both muscles from D-W. However, that is far from the observed in WKY and SHR, in which diabetes reduced GLUT4 protein only in EDL from SHR; even so, the protein content remained much higher in D-SHR than in D-W. This may hold the key explanation for the glycemic values observed in D-WKY and D-SHR: the increased GLUT4 content in skeletal muscle improves insulin-induced glucose disposal, and this allows similar insulinemic levels to achieve glycemic levels ~50% lower than those observed in D-W.

It has been proposed that the diabetes effect on reducing *Slc2a4*/GLUT4 expression is caused by the hyperglycemia *per se*, since the glycemia reduction by phlorizin restored the GLUT4 expression in diabetic rats [25]. Therefore, it is possible that *Slc2a4* and GLUT4 expression are much more affected in D-W rats than in D-WKY and D-SHR because of their severe hyperglycemia. Conversely, increased GLUT4 expression decreases glycemia [5], and D-WKY and D-SHR showed much more GLUT4. Thus, both mechanisms seems to establish a virtuous circle, in which decreased glycemia enhances GLUT4, and increased GLUT4 decreases glycemia, leading to an improved glycemic homeostasis.

As highlighted above, discrepancy between changes in *Slc2a4* mRNA and GLUT4 protein implies posttranscriptional regulation, and this is somewhat common for the *Slc2a4* gene. Our group has found discrepancies between *Slc2a4* mRNA and GLUT4 protein in skeletal muscles, with variations in the *Slc2a4* mRNA poly(A) tail length, explaining the altered translation efficiency [13, 14]. Here, discrepancies were detected independently of increase or decrease of the *Slc2a4* mRNA, and the data suggest that both WKY and SHR need more mRNA transcripts to maintain proper GLUT4 level.

Finally, we point out that WKY and SHR similarly regulate some metabolic-related parameters, such as body weight, diabetes-induced impairment of glycemia and *Slc2a4*/GLUT4 regulations, all of them different from W rats. This feature can disable WKY as a good control for SHR, at least in studies focusing on glucose homeostasis. In fact, for some other features, such as cardiomyocyte hypertrophy and the left ventricular diastolic stiffness, WKY rats were much closer to SHR than to W rats [4].

## Conclusion

Concluding, the present study reveals that, although the insulin resistance was observed only in SHR, both WKY and SHR similarly differ from W rats in terms of reduced body weight and increased *Slc2a4*/GLUT4 expression in skeletal muscles. Furthermore, diabetes induction promoted a much smaller impairment of glycemic homeostasis in WKY and SHR, and this was not related to different reduction in plasma insulin concentration. Importantly, diabetes did not reduce *Slc2a4*/GLUT4 expression in muscles, as expected. In summary, when compared to W rats, both WKY and SHR are similarly protected against severe hyperglycemia, through a pathway unrelated to commitment of insulin secretion, but apparently involving absence of suppression of *Slc2a4/*GLUT4 expression.

## References

[1] Okamoto K, Aoki K (1963). Development of a strain of spontaneously hypertensive rats. Jpn Circ J.

[2] Kurtz TW, Morris RC (1987). Biological variability in Wistar-Kyoto rats: Implications for research with the spontaneously hypertensive rat. Hypertension.

[3] Katayama S, Inaba M, Maruno Y, Morita T, Awata T, Oka Y (1997). Glucose intolerance in spontaneously hypertensive and Wistar-Kyoto rats: enhanced gene expression and synthesis of skeletal muscle glucose transporter 4. Hypertens Res.

[4] Aiello EA, Villa-Abrille MC, Escudero EM, Portiansky EL, Pérez NG, Hurtado MCC, Cingolani HE (2004). Myocardial hypertrophy of normotensive Wistar-Kyoto rats. Am J Physiol Heart Circ Physiol.

[5] Corrêa-Giannella ML, Machado UF (2013). SLC2A4 gene: a promising target for pharmacogenomics of insulin resistance. Pharmacogenomics.

[6] Paternostro G, Clarke K, Heath J, Seymour AM, Radda GK (1995). Decreased GLUT-4 mRNA content and insulin-sensitive deoxyglucose uptake show insulin resistance in the hypertensive rat heart. Cardiovasc Res.

[7] Reaven GM (2006). The metabolic syndrome: is this diagnosis necessary?. Am J Clin Nutr.

[8] UK Prospective Diabetes Study Group (1998). Tight blood pressure control and risk of macrovascular and microvascular complications in type 2 diabetes: UKPDS 38. BMJ.

[9] Schaan BD, Irigoyen MC, Bertoluci MC, Lima NG, Passaglia J, Hermes E, Oliveira FR, Okamoto M, Machado UF (2005). Increased urinary TGF-beta1 and cortical renal GLUT1 and GLUT2 levels: additive effects of hypertension and diabetes. Nephron Physiol.

[10] Sabino-Silva R, Alves-Wagner ABT, Burgi K, Okamoto MM, Alves AS, Lima GA, Freitas HS, Antunes VR, Machado UF (2010). SGLT1 protein expression in plasma membrane of acinar cells correlates with the sympathetic outflow to salivary glands in diabetic and hypertensive rats. Am J Physiol Endocrinol Metab.

[11] Vestri S, Okamoto MM, Freitas HS, Aparecida dos Santos R, Nunes MT, Morimatsu M, Heimann JC, Machado UF (2001). Changes in sodium or glucose filtration rate modulate expression of glucose transporters in renal proximal tubular cells of rat. J Membr Biol.

[12] Patinha D, Afonso J, Sousa T, Morato M, Albino-Teixeira A (2014). Activation of adenosine receptors improves renal antioxidant status in diabetic Wistar but not SHR rats. Ups J Med Sci.

[13] Seraphim PM, Nunes MT, Giannocco G, Machado UF (2007). Age related obesity-induced shortening of GLUT4 mRNA poly(A) tail length in rat gastrocnemius skeletal muscle. Mol Cell Endocrinol.

[14] Alves-Wagner ABT, Freitas HS, Souza PB, Seraphim PM, Mori RCT, Machado UF (2009). β-adrenergic activity preserves GLUT4 protein in glycolytic fibers in fasting. Muscle Nerve.

[15] Ferguson RE, Carroll HP, Harris A, Maher ER, Selby PJ, Banks RE (2005). Housekeeping proteins: a preliminary study illustrating some limitations as useful references in protein expression studies. Proteomics.

[16] Papa PC, Seraphim PM, Machado UF (1997). Loss of weight restores GLUT 4 content in insulin-sensitive tissues of monosodium glutamate-treated obese mice. Int J Obes Relat Metab Disord.

[17] Freitas HS, Anhê GF, Melo KF, Okamoto MM, Oliveira-Souza M, Bordin S, Machado UF (2008). Na(+) -glucose transporter-2 messenger ribonucleic acid expression in kidney of diabetic rats correlates with glycemic levels: involvement of hepatocyte nuclear factor-1alpha expression and activity. Endocrinology.

[18] Sabino-Silva R, Freitas HS, Lamers ML, Okamoto MM, Santos MF, Machado UF (2009). Na + -glucose cotransporter SGLT1 protein in salivary glands: potential involvement in the diabetes-induced decrease in salivary flow. J Membr Biol.

[19] Okamoto MM, Anhê GF, Sabino-Silva R, Marques MF, Freitas HS, Mori RC, Melo KF, Machado UF (2011). Intensive insulin treatment induces insulin resistance in diabetic rats by imparing glucose metabolism-related mechanisms in muscle and liver. J Endocrinol.

[20] Baron AD, Brechtel G, Wallace P, Edelman SV (1988). Rates and tissue sites of non-insulin- and insulin-mediated glucose uptake in humans. Am J Physiol.

[21] Zecchin HG, Bezerra RM, Carvalheira JB, Carvalho-Filho MA, Metze K, Franchini KG, Saad MJ (2003). Insulin signaling pathways in aorta and muscle from two animal models of insulin resistance - the obese middle-aged and the spontaneously hypertensive rats. Diabetologia.

[22] Chiappe De Cingolani GE, Caldiz CI (2004). Insulin resistance and GLUT-4 glucose transporter in adipocytes from hypertensive rats. Metabolism.

[23] Cabassi A, Vinci S, Cantoni AM, Quartieri F, Maschini L, Cavazzini S, Cavatorta A, Borghetti A (2002). Sympathetic activation in adipose tissue and skeletal muscle of hypertensive rats. Hypertension.

[24] Mora S, Pessin JE (2000). The MEF2A isoform is required for striated muscle-specific expression of the insulin-responsive GLUT4 glucose transporter. J Biol Chem.

[25] Dimitrakoudis D, Ramlal T, Rastogi S, Vranic M, Klip A (1992). Glycaemia regulates the glucose transporter number in the plasma membrane of rat skeletal muscle. Biochem J.

